# Dual-Phase β-Amyloid PET Captures Neuronal Injury and Amyloidosis in Corticobasal Syndrome

**DOI:** 10.3389/fnagi.2021.661284

**Published:** 2021-05-13

**Authors:** Julia Schmitt, Carla Palleis, Julia Sauerbeck, Marcus Unterrainer, Stefanie Harris, Catharina Prix, Endy Weidinger, Sabrina Katzdobler, Olivia Wagemann, Adrian Danek, Leonie Beyer, Boris-Stephan Rauchmann, Axel Rominger, Mikael Simons, Peter Bartenstein, Robert Perneczky, Christian Haass, Johannes Levin, Günter U. Höglinger, Matthias Brendel, the German Imaging Initiative for Tauopathies (GII4T)

**Affiliations:** ^1^Department of Nuclear Medicine, University Hospital of Munich, LMU Munich, Munich, Germany; ^2^Department of Neurology, University Hospital of Munich, LMU Munich, Munich, Germany; ^3^Department of Psychiatry and Psychotherapy, University Hospital, LMU Munich, Munich, Germany; ^4^Department of Radiology, University Hospital of Munich, LMU Munich, Munich, Germany; ^5^Department of Nuclear Medicine, University of Bern, Inselspital, Bern, Switzerland; ^6^Munich Cluster for Systems Neurology (SyNergy), Munich, Germany; ^7^German Center for Neurodegenerative Diseases (DZNE), Munich, Germany; ^8^Institute of Neuronal Cell Biology, Technical University Munich, Munich, Germany; ^9^Ageing Epidemiology Research Unit (AGE), School of Public Health, Imperial College London, London, United Kingdom; ^10^Faculty of Medicine, Chair of Metabolic Biochemistry, Biomedical Center (BMC), Ludwig-Maximilians-Universtität München, Munich, Germany; ^11^Department of Neurology, Medizinische Hochschule Hannover, Hanover, Germany; ^12^Department of Neurology, Technical University Munich, Munich, Germany

**Keywords:** amyloid, PET, dual phase, neuronal injury, corticobasal syndrome

## Abstract

**Objectives:** In recent years several ^18^F-labeled amyloid PET (Aβ-PET) tracers have been developed and have obtained clinical approval. There is evidence that Aβ-PET perfusion can provide surrogate information about neuronal injury in neurodegenerative diseases when compared to conventional blood flow and glucose metabolism assessment. However, this paradigm has not yet been tested in neurodegenerative disorders with cortical and subcortical affection. Therefore, we investigated the performance of early acquisition ^18^F-flutemetamol Aβ-PET in comparison to ^18^F-fluorodeoxyglucose (FDG)-PET in corticobasal syndrome (CBS).

**Methods:** Subjects with clinically possible or probable CBS were recruited within the prospective Activity of Cerebral Networks, Amyloid and Microglia in Aging and Alzheimer’s Disease (ActiGliA) observational study and all CBS cases with an available FDG-PET prior to Aβ-PET were selected. Aβ-PET was acquired 0–10 min p.i. (early-phase) and 90–110 min p.i. (late-phase) whereas FDG-PET was recorded statically from 30 to 50 min p.i. Semiquantitative regional values and asymmetry indices (AI) were compared between early-phase Aβ-PET and FDG-PET. Visual assessments of hypoperfusion and hypometabolism were compared between both methods. Late-phase Aβ-PET was evaluated visually for assessment of Aβ-positivity.

**Results:** Among 20 evaluated patients with CBS, 5 were Aβ-positive. Early-phase Aβ-PET and FDG-PET SUVr correlated highly in cortical (mean *R* = 0.86, range 0.77–0.92) and subcortical brain regions (mean *R* = 0.84, range 0.79–0.90). Strong asymmetry was observed in FDG-PET for the motor cortex (mean |AI| = 2.9%), the parietal cortex (mean |AI| = 2.9%), and the thalamus (mean |AI| = 5.5%), correlating well with AI of early-phase Aβ-PET (mean *R* = 0.87, range 0.62–0.98). Visual assessments of hypoperfusion and hypometabolism were highly congruent.

**Conclusion:** Early-phase Aβ-PET facilitates assessment of neuronal injury in CBS for cortical and subcortical areas. Known asymmetries in CBS are captured by this method, enabling assessment of Aβ-status and neuronal injury with a single radiation exposure at a single visit.

## Introduction

Corticobasal syndrome (CBS) is a movement disorder with clinical atypical Parkinsonism and additional cognitive impairment (Alexander et al., 2014; van Eimeren et al., 2019). Neuropathologically, patients with clinical CBS reveal a high variability of underlying protein misfolding, including four-repeat tauopathies, TDP43-positive fronto-temporal dementia, disease with Lewy-bodies and Alzheimer’s disease (AD) (Parmera et al., 2016). The largest autopsy-controlled study found corticobasal degeneration (CBD) in 35% of cases, followed by AD in 23%, progressive supranuclear palsy in 13%, and TDP43-positive frontotemporal lobar degeneration in 13% of the studied cases (Lee et al., 2011). Thus, while the majority of CBS patients are characterized by a four repeat (4R) tauopathy there are also relevant numbers of patients with concomitant 3/4R tau and β-amyloid (Aβ) pathology (Rosler et al., 2019). In this regard, the frequency of AD neuropathology in clinical CBS varies among different autopsy cohorts (Parmera et al., 2016), ranging from 10 to 50%. In spite of intensive research, at present, there is no accepted causal treatment for CBS; the available symptomatic treatments are of limited efficacy and are supported only by low-level evidence (Levin et al., 2016). Assessment of different underlying neuropathologies in neurodegenerative disorders *in vivo* will be necessary to stratify patients into future targeted personalized therapies (Coughlin and Irwin, 2017), and quantification of neuronal injury can serve as an objective progression biomarker. ^18^F-fluorodeoxyglucose positron-emission-tomography (FDG)-PET can be used to detect neuronal injury in CBS, like in AD (Jack et al., 2018), and is already implemented in current Movement Disorders Society (MDS) diagnosis criteria (Höglinger et al., 2017). However, FDG-PET has limited specificity for the causal neuropathology despite facilitating differential diagnosis between neurodegenerative disorders when using it in clinical routine settings (Bloudek et al., 2011). Aβ-PET is a powerful tool to detect fibrillar Aβ plaques *in vivo* (Sabri et al., 2015) and also performs well in detecting Aβ-positive CBS (Ossenkoppele et al., 2015). Perfusion phase Aβ-PET was already evaluated in diagnostic workup of neurodegenerative disorders as a surrogate for neuronal injury and potential substitute of FDG-PET. Several recent studies have shown comparable reductions of Aβ-PET perfusion and metabolic deficits in PET using FDG (Meyer et al., 2011; Hsiao et al., 2012). Our previous study examined the clinical use of ^18^F-Florbetaben PET by additional visual interpretation of early-phase acquisitions and indicated strong visual and quantitative correlations between ^18^F-Florbetaben perfusion and glucose metabolism, irrespective of the Aβ status (Daerr et al., 2017). Other groups likewise concluded that early-phase Aβ-PET (^11^C-PiB, ^18^F-Florbetaben) can be used to quantify neuronal injury (Chen et al., 2015; Tiepolt et al., 2016). However, this paradigm has not yet been tested in neurodegenerative disorders with cortical and subcortical affection.

Hence, we sought to evaluate the performance of early acquisition ^18^F-flutemetamol Aβ-PET in comparison to FDG-PET in CBS. We correlated quantitative regional values of both methods in cortical and subcortical brain areas and we compared the ability of both methods to detect known asymmetries of neuronal injury in CBS.

## Materials and Methods

### Study Design

Patients with a diagnosis of clinically possible or probable CBS according to current MDS diagnostic criteria (Höglinger et al., 2017) or the Armstrong criteria (Armstrong et al., 2013) were recruited within the prospective Activity of Cerebral Networks, Amyloid and Microglia in Aging and Alzheimer’s Disease (ActiGliA) observation study. Recruiting sites were located at the department of Neurology and the department of Psychiatry and Psychotherapy at the Ludwig-Maximilians-Universität Munich. ActiGliA is a prospective cohort study of the Munich Cluster for Systems Neurology (SyNergy) at Ludwig-Maximilians-University in Munich, Germany, initiated in 2017 that comprises comprehensive clinical assessment, multimodal prospective imaging *in vivo* and fluid biomarker analyses in AD spectrum and CBS patients and controls, approved by the ethics committee of the Ludwig-Maximilians-University Munich (project numbers 17-755 and 17-569) in line with the declaration of Helsinki. All patients gave their written informed consent. We selected all CBS patients with an FDG-PET scan at the Department of Nuclear Medicine prior to study inclusion (maximum time gap 1 year). Dual phase ^18^F-flutemetamol Aβ-PET was performed within ActiGliA. Disease duration was recorded as the time from symptom onset to the midpoint of FDG-PET and Aβ-PET.

### Radiosynthesis and PET Imaging

^18^F-FDG was purchased commercially and FDG-PET was performed in compliance with to the EANM protocol (Boellaard et al., 2015). 137 ± 14 MBq ^18^F-FDG were administered after fasting for at least 6 h and patient preparation ≥20 min with standardized reduction of noise and visual input. Emission recording was performed 30–50 min p.i. with a Biograph 64 PET/CT system (Siemens Healthcare, Erlangen, Germany) after performing a low dose CT scan for attenuation correction. Iterative reconstruction of a single 20 min frame was performed with three-dimensional ordered-subset expectation maximization/3-D maximum *a posteriori* using four and 21 iterations, respectively. Final voxel-size in the 336 × 336 × 109 matrix was 1.0 × 1.0 × 2.0 mm^3^.

^18^F-flutemetamol was synthesized as described earlier (Senda et al., 2015). ^18^F-flutemetamol Aβ-PET was performed with dual phase 0–10 min (early-phase) and 90–110 min (late-phase) emission recordings after administration of 187 ± 10 MBq ^18^F-flutemetamol at the same PET/CT system and with equal reconstruction parameter. The low dose CT scan was performed prior to the late-phase acquisition and this scan also used for attenuation correction of the early-phase data. In a subset of patients with CBS (*n* = 12), 1-min frame reconstructions of early-phase Aβ-PET were performed for a dedicated time-window evaluation.

### PET Imaging Analysis

#### Preprocessing

For spatial normalization, early-phase and late-phase Aβ-PET templates and an FDG-PET template were created in the Montreal Neurology Institute (MNI) space using the PMOD software (version 3.9, PMOD Technologies Ltd., Zurich, Switzerland). All templates were generated using 20 randomly selected ActiGliA cases with structural T1-weighted MRI. PET images were rigidly matched to the MRI image, fused and the alignment was controlled by visual assessment. The individual MRI was co-registered to a T1 MRI template in the MNI space by non-linear warping and both transformations were connected to minimize interpolation. Single images were averaged to templates after global mean normalization. All evaluated PET data of this investigation were co-registered to the matching PET templates by non-linear warping and controlled by visual assessment, as described previously (Hsiao et al., 2013).

#### Analysis of FDG-PET and Early-Phase Aβ-PET

**FIGURE 1:**
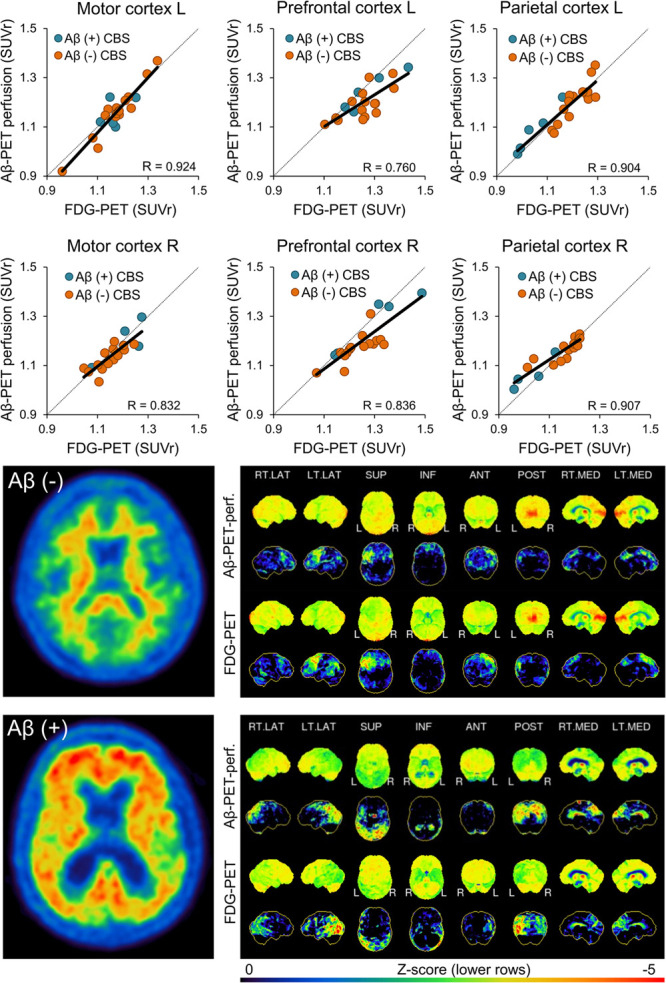
Agreement between early phase Aβ-PET and FDG-PET for cortical regions. Plots in the upper two rows show correlation of both modalities for regions with strongest neuronal injury. Images in the lower two rows illustrate patient examples for Aβ-negative and Aβ-positive cases. The left image shows an axial slice of the late-phase Aβ-PET and the right panel depicts 3DSSP surface projections for early-phase Aβ-PET and FDG-PET. The color scale of late phase Aβ-PET images was set to 90% of the pons signal intensity. SUVr, standardized-uptake-value-ratios; FDG-PET, ^18^F-fluorodesoxyglucose positron-emission-tomography; SD, standard deviation; L, left; R, right; LAT, lateral; SUP, superior; INF, inferior; ANT, anterior; POST, posterior; MED, medial; Aβ, β-amyloid; *R*, Pearson’s coefficient of correlation; CBS, corticobasal syndrome.

**FIGURE 2:**
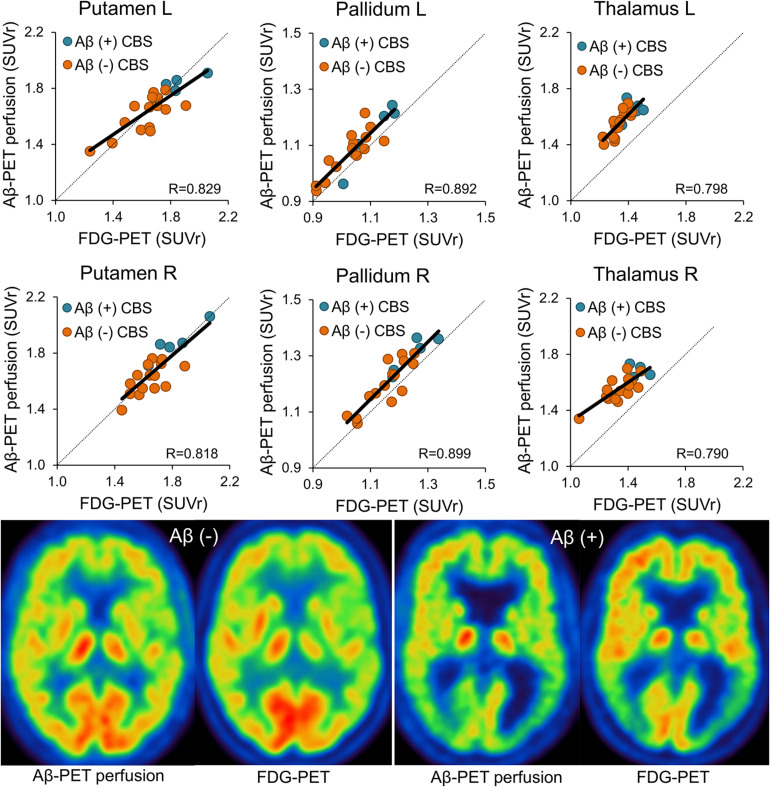
Agreement between early phase Aβ-PET and FDG-PET for subcortical regions. Plots in the upper two rows show correlation of both modalities for all subcortical regions. Images in the lower row illustrate patient examples for Aβ-negative and Aβ-positive cases. Images show corresponding axial planes for early-phase Aβ-PET and FDG-PET. The color scale of early phase Aβ-PET and FDG-PET images was set to the maximum signal intensity. SUVr, standardized-uptake-value-ratios; FDG-PET, ^18^F-fluorodesoxyglucose positron-emission-tomography; SD, standard deviation; L, left; R, right; LAT, lateral; SUP, superior; INF, inferior; ANT, anterior; POST, posterior; MED, medial; Aβ, β-amyloid; *R*, Pearson’s coefficient of correlation; CBS, corticobasal syndrome.

Predefined volumes of interest (VOIs) of the Hammer Atlas (Hammers et al., 2003) were applied, encoding 83 brain areas in the stereotactic MNI space. Target regions of the 83 cortical and subcortical brain areas were defined as follows and separately for the left and right hemisphere: motor cortex, prefrontal cortex, parietal cortex, putamen, thalamus and pallidum. For activity normalization, we used whole cerebellum (CBL) or whole brain (=global mean; GLM) scaling of all target regions to generate SUVr_*CBL*_ and SUVr_*GLM*_ images. Asymmetry of SUVr was assessed by calculation of the asymmetry index AI = (*L* − *R*)/(*L* + *R*) (Vernaleken et al., 2007) for all target regions. FDG-PET and early-phase Aβ-PET images were also processed by three-dimensional stereotactic surface projection (Minoshima et al., 1995) using the standard in house FDG-PET normal cohort (Beyer et al., 2018) as already established for early-phase ^18^F-florbetaben (Daerr et al., 2017). The normal cohort was matched for age (67 ± 6) and sex (10 female, 14 male). Two expert and two experienced readers graded the cortical target regions into no (=0), mild (=1), intermediate (=2) or severe (=3) hypoperfusion/-metabolism. Readers were trained to rate z-scores between 2 and 3 as mild (green presentation), z-scores between 3 and 4 as intermediate (yellow to orange presentation), and z-scores between 4 and 5 as severe (orange to red presentation), as described previously (Beyer et al., 2018; Kreuzer et al., 2021). The reader was blind to the modality and all surface projections were provided in a digital print format. Axial slices through the basal ganglia (maximum intensity scaling) served for equal scoring of the striatum and the thalamus. For subcortical regions, the readers were trained to take an FDG-PET template of a normal cohort into consideration for the visual judgment. All axial slices (*n* = 4 per subject, standardized localization) were extracted after spatial normalization via the Hermes software package (FDG-PET viewer, V4.17, HERMES medical solutions AD, Stockholm, Sweden). Examples of the three-dimensional stereotactic surface projections and the axial slices used for the visual read are provided in Figure 1 and Figure 2, whereas details of the axial slices are provided in Supplementary Figure 1.

#### Analysis of Late-Phase Aβ-PET

Visual assessment was performed by a single expert reader (blinded to the subject’s identity and radiotracer used) resulting in a binary decision of positive or negative Aβ status.

### Statistics

SPSS (V.25, IBM statistics, New York) was used for all statistical testing. Normal distribution of SUVr values was evaluated by a Kolmogorov-Smirnov test. Mean regional SUVr were calculated for FDG-PET and early-phase Aβ-PET. Pearson correlation coefficients (*R*) were calculated between FDG-PET and early-phase Aβ-PET SUVr in all target regions and for AI of all target regions. Levels of correlation in all target regions were compared between global mean and cerebellar normalization by a paired *t*-test, after Fisher’s z-transformation. In the subsample of 12 cases with 1-min frame reconstruction of early-phase Aβ-PET, SUVr values of each frame were correlated with SUVr values of FDG-PET in order to test the agreement between both modalities as a function of the early-phase Aβ-PET acquisition time. Interclass correlation coefficients (intra-reader and inter-reader) were calculated to test for agreement between regional grading of surface projections and axial basal ganglia slices. A two-way mixed model was applied by measuring the absolute agreement with a confidence interval of 95%. *p* values < 0.05 were considered significant.

## Results

### Demographics, Clinical Data and Clinical Routine Assessment of FDG-PET

A total of 20 subjects (nine male, 11 female) were included in the study (Table 1). All Aβ-negative patients fulfilled a clinical diagnosis of CBS according to current MDS criteria (Höglinger et al., 2017). All Aβ-positive CBS patients fulfilled the Armstrong criteria (Armstrong et al., 2013). Five out of 20 late-phase ^18^F-flutemetamol Aβ-PETs were visually classified as Aβ-positive. 14 out of 20 FDG-PETs were classified to show a significant neuronal injury in clinical routine assessment. Most frequently affected cortical regions were the motor cortex (65%), the parietal cortex (60%), and the prefrontal cortex (50%). Subcortical regions revealed abnormalities in 30% and 70% for striatum and thalamus.

**TABLE 1:** Demographics of the study population.

Study groups	*N*	Age (y ± SD)	Sex (m/f)	Disease severity (PSPRS)	Cognition (MoCA)	Disease duration (months ± SD)	Difference between FDG-PET and Aβ-PET (months ± SD)
All patients with CBS	20	68 ± 9	9/11	28 ± 14	20 ± 8	28 ± 22	4.9 ± 7.8
Aβ-positive CBS	5	69 ± 8	3/2	29 ± 10	10 ± 5	35 ± 27	5.7 ± 7.1
Aβ-negative CBS	15	68 ± 10	6/9	28 ± 16	24 ± 4	26 ± 21	4.7 ± 8.1

*CBS, corticobasal syndrome; m, male; f, female; y, year; SD, standard deviation; Aβ, β-amyloid; MoCA, Montreal Cognitive Assessment; PSPRS, Progressive Supranuclear Palsy Rating Scale; FDG-PET, ^18^F-fluorodesoxyglucose positron-emission-tomography.*

### VOI-Based Comparison of Early-Phase ^18^F-Flutemetamol Aβ-PET and FDG-PET

**FIGURE 3:**
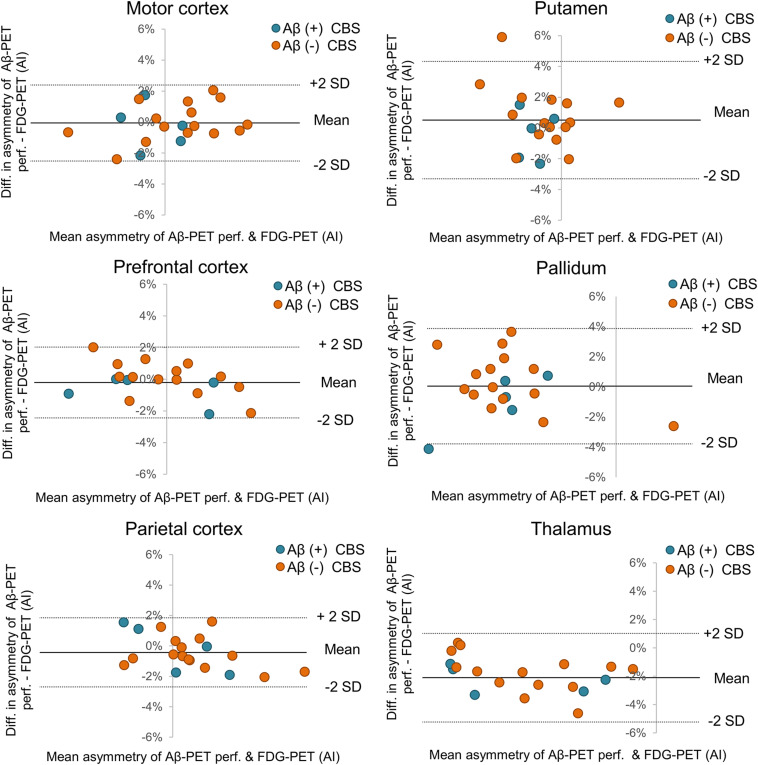
Bland-Altman plot of asymmetry agreement between early phase Aβ-PET and FDG-PET. Cortical regions with strongest asymmetry in FDG-PET (left column) and subcortical regions (right column) are visualized with respect to individual agreement of asymmetry indices (AI). FDG-PET, ^18^F-fluorodesoxyglucose positron-emission-tomography; SD, standard deviation; Aβ, β-amyloid; *R*, Pearson’s coefficient of correlation; CBS, corticobasal syndrome.

Single frames of early-phase Aβ-PET indicated a constant SUVr agreement with FDG-PET over the 10-min acquisition time of early-phase Aβ-PET (Supplementary Figure 2). Thus, the full 10-min Aβ-PET early-phase acquisition was used for all subsequent analyses. Early-phase Aβ-PET and FDG-PET SUVr correlated highly in cortical brain regions (mean *R* = 0.86, range 0.77–0.92, all *p* ≤ 0.001; Figure 1 when using global mean scaling. Importantly, a strong agreement between early-phase Aβ-PET and FDG-PET SUVr was also observed in subcortical areas (mean *R* = 0.84, range 0.79–0.90, all *p* < 0.001; Figure 2), when using global mean scaling. Cerebellar normalization mirrored the results of global mean scaling at a slightly lower level of correlation (mean *R* values: 0.84 ± 0.08 vs. 0.76 ± 0.12; paired *t*-test after Fisher’s z-transformation: *p* = 0.013). Results of individual regions are reported in Table 2. Strong asymmetry was observed in FDG-PET for central (mean |AI| = 2.89%) and parietal (mean |AI| = 2.85%) cortices as well as the thalamus (mean |AI| = 5.45%). AI of early-phase Aβ-PET correlated highly with AI observed in FDG-PET (mean *R* = 0.87, range 0.62–0.98). Agreements of AI measured by early-phase Aβ-PET and FDG-PET are visualized as Bland-Altman plots in Figure 3.

**TABLE 2:** Semiquantitative results of early-phase Aβ-PET and FDG-PET.

	Global mean normalization					Cerebellar normalization				
	Aβ-PET perfusion (SUVr)		FDG-PET (SUVr)		*R* (*p*)	Aβ-PET perfusion (SUVr)		FDG-PET (SUVr)		*R* (*p*)
	Mean	SD	Mean	SD		Mean	SD	Mean	SD	
Motor cortex L	1.158	0.095	1.173	0.078	0.924 (0.000)	0.959	0.125	1.076	0.127	0.868 (0.000)
Motor cortex R	1.142	0.060	1.156	0.062	0.832 (0.000)	0.943	0.087	1.060	0.101	0.733 (0.000)
Prefrontal cortex L	1.206	0.067	1.261	0.079	0.760 (0.000)	0.995	0.086	1.156	0.115	0.619 (0.004)
Prefrontal cortex R	1.199	0.084	1.249	0.092	0.836 (0.000)	0.989	0.085	1.145	0.125	0.667 (0.001)
Parietal cortex L	1.176	0.092	1.172	0.092	0.904 (0.000)	0.975	0.095	1.078	0.150	0.844 (0.000)
Parietal cortex R	1.148	0.062	1.137	0.083	0.907 (0.000)	0.949	0.093	1.045	0.140	0.859 (0.000)
Temporal cortex L	1.149	0.071	1.138	0.073	0.967 (0.000)	0.975	0.095	1.051	0.109	0.907 (0.000)
Temporal cortex R	1.126	0.048	1.108	0.036	0.764 (0.000)	0.949	0.093	1.015	0.079	0.852 (0.000)
Occipital cortex L	1.244	0.081	1.211	0.085	0.839 (0.000)	1.373	0.129	1.110	0.120	0.911 (0.000)
Occipital cortex R	1.237	0.050	1.193	0.060	0.637 (0.003)	1.383	0.119	1.094	0.109	0.867 (0.000)
Putamen L	1.666	0.147	1.680	0.174	0.929 (0.000)	1.373	0.129	1.535	0.154	0.640 (0.002)
Putamen R	1.679	0.153	1.682	0.141	0.818 (0.000)	1.383	0.119	1.537	0.125	0.567 (0.009)
Pallidum L	1.092	0.089	1.049	0.079	0.892 (0.000)	0.900	0.078	0.959	0.077	0.770 (0.000)
Pallidum R	1.225	0.090	1.178	0.079	0.899 (0.000)	1.009	0.076	1.077	0.083	0.733 (0.000)
Thalamus L	1.570	0.095	1.356	0.073	0.798 (0.000)	1.294	0.088	1.240	0.083	0.627 (0.003)
Thalamus R	1.573	0.096	1.363	0.109	0.790 (0.000)	1.296	0.084	1.247	0.120	0.668 (0.001)

*All regional standardized-uptake-value-ratios (SUVr) values are given for global mean normalization and cerebellar scaling together with their correlation coefficient. FDG-PET, ^18^F-fluorodesoxyglucose positron-emission-tomography; *R*, Pearson’s coefficient of correlation; SD, standard deviation; L, left; R, right.*

### Visual 3D-SSP Comparison of Early-Phase ^18^F-Flutemetamol Aβ-PET and FDG-PET

Visual assessment of target regions showed high ICC between early-phase ^18^F-flutemetamol Aβ-PET and FDG-PET for intra-reader agreement among all four readers (mean ICC for all evaluated regions: 0.86 ± 0.10; 0.85 ± 0.09; 0.81 ± 0.12; and 0.75 ± 0.10; Table 3). There was no remarkable difference between experienced and expert readers. Inter-reader agreement indicated high ICC for early-phase Aβ-PET (mean ICC for all evaluated regions: 0.91 ± 0.06) which was comparable to inter-reader ICC for FDG-PET (mean ICC for all evaluated regions: 0.92 ± 0.06). This was also resembled by a mixed ICC including the four ratings of early-phase Aβ-PET and the four ratings of FDG-PET (mean ICC for all evaluated regions: 0.93 ± 0.04).

**TABLE 3:** Visual comparison between surface projections of early-phase Aβ-PET and FDG-PET.

Region	Expert 1 (intra-rater ICC, 95%CI)	Expert 2 (Intra-rater ICC, 95%CI)	Experienced 1 (intra-rater ICC, 95%CI)	Experienced 2 (intra-rater ICC, 95%CI)	FDG-PET (inter-rater ICC, 95%CI)	Aβ-PET perfusion (inter-rater ICC, 95%CI)	Combined (inter-rater ICC, 95%CI)
Motor cortex L	0.916 (0.790–0.966)	0.742 (0.368–0.897)	0.905 (0.760–0.962)	0.864 (0.655–0.946)	0.906 (0.808–0.959)	0.864 (0.732–0.940)	0.933 (0.878–0.970)
Motor cortex R	0.942 (0.812–0.979)	0.808 (0.526–0.923)	0.928 (0.818–0.971)	0.758 (0.397–0.904)	0.858 (0.720–0.938)	0.850 (0.703–0.934)	0.834 (0.698–0.924)
Prefrontal cortex L	0.811 (0.524–0.925)	0.749 (0.385–0.899)	0.627 (0.144–0.849)	0.519 (−0.180–0.807)	0.969 (0.939–0.986)	0.867 (0.736–0.942)	0.932 (0.876–0.969)
Prefrontal cortex R	0.950 (0.874–0.980)	0.806 (0.513–0.923)	0.901 (0.750–0.961)	0.882 (0.702–0.953)	0.979 (0.959–0.991)	0.932 (0.867–0.970)	0.972 (0.948–0.987)
Parietal cortex L	0.902 (0.519–0.969)	0.864 (0.428–0.955)	0.915 (0.285–0.977)	0.898 (0.483–0.968)	0.975 (0.952–0.989)	0.982 (0.964–0.992)	0.977 (0.955–0.990)
Parietal cortex R	0.895 (0.564–0.965)	0.824 (0.253–0.943)	0.848 (0.154–0.955)	0.856 (0.008–0.961)	0.989 (0.978–0.995)	0.964 (0.929–0.984)	0.972 (0.942–0.988)
Temporal cortex L	0.938 (0.835–0.976)	0.912 (0.772–0.965)	0.872 (0.683–0.949)	0.831 (0.577–0.933)	0.968 (0.938–0.986)	0.973 (0.946–0.988)	0.976 (0.956–0.989)
Temporal cortex R	0.772 (0.441–0.909)	0.851 (0.622–0.941)	0.846 (0.512–0.944)	0.883 (0,0.88–0.955)	0.952 (0.905–0.979)	0.970 (0.941–0.987)	0.962 (0.931–0.983)
Occipital cortex L	0.907 (0.715–0.966)	0.537 (−0.074–0.810)	0.750 (0.386–0.900)	0.828 (0.574–0.931)	0.923 (0.841–0.967)	0.910 (0.823–0.960)	0.936 (0.881–0.971)
Occipital cortex R	0.858 (0.631–0.945)	0.714 (0.263–0.888)	0.720 (0.319–0.888)	0.574 (–0.034–0.829)	0.859 (0.719–0.938)	0.937 (0.874–0.972)	0.911 (0.836–0.960)
Striatum L	0.859 (0.632–0.945)	0.705 (0.282–0.881)	0.892 (0.725–0.957)	0.897 (0.741–0.959)	0.947 (0.981–0.977)	0.849 (0.655–0.937)	0.939 (0.886–0.973)
Striatum R	0.579 (−0.22–0.831)	0.730 (0.322–0.893)	0.898 (0.743–0.960)	0.762 (0.397–0.906)	0.803 (0.592–0.915)	0.770 (0.481–0.904)	0.868 (0.750–0.940)
Thalamus L	0.621 (0.061–0.849)	0.617 (0.071–0.846)	0.843 (0.612–0.937)	0.864 (0.661–0.946)	0.843 (0.668–0.933)	0.922 (0.845–0.966)	0.917 (0.845–0.963)
Thalamus R	0.821 (0.557–0.929)	0.634 (0.116–0.853)	0.925 (0.813–0.970)	0.900 (0.748–0.961)	0.898 (0.790–0.956)	0.916 (0.832–0.963)	0.941 (0.889–0.973)

*Intraclass correlation coefficients (ICC) are given for intra-rater (early-phase Aβ-PET vs. FDG-PET) and inter-rater (early-phase Aβ-PET/FDG-PET/combined) comparisons together with their 95% confidence interval (CI). L, left; R, right; FDG-PET, ^18^F-fluorodesoxyglucose positron-emission-tomography.*

## Discussion

We present the first investigation comparing early-phase Aβ-PET and FDG-PET for the assessment of neuronal injury in CBS. The well-defined prospective cohort of this rare neurodegenerative disorder allowed to test for the value of perfusion phase Aβ-PET in neurodegeneration of subcortical and cortical brain areas. Keeping potential temporal gaps in the sensitivity of both modalities in mind, our data revealed that dynamic or dual time point imaging of Aβ-PET can deliver a similar information of neuronal injury when compared to FDG-PET in patients with CBS. Furthermore, asymmetric neuronal injury, representing the imaging correlate of clinical asymmetry in CBS, was also captured sufficiently by early-phase Aβ-PET.

The value of the propagated dual phase imaging technique is especially high in CBS, since underlying Aβ pathology is frequently found (23%) in autopsy studies and has associations with the clinical phenotype (Lee et al., 2011). Thus, the information of Aβ-positivity can help to judge clinical symptoms of AD-CBS correctly which implicates an increase in the overall accuracy of *ante mortem* diagnosis of CBS. Our cohort comprised 25% Aβ-positive CBS cases which is in line with 23% AD pathology at autopsy (Lee et al., 2011). We acknowledge that the provided sample size is small, and we note that the frequency of underlying AD neuropathology varies among different CBS autopsy studies (Parmera et al., 2016). Thus, the reported frequency of Aβ-positivity in our cohort could be biased by both the limited number of patients and the overall variability. We did not put a focus on neuronal injury differences between Aβ-positive and Aβ-negative CBS cases, but qualitatively there was an increased involvement of parieto-temporal regions in Aβ-positive CBS cases and a more severe affection of subcortical regions in Aβ-negative CBS cases (see Figures 1, 2), consistent with recently reported FDG-PET patterns in autopsy proven subtypes of CBS (Pardini et al., 2019). These observations also fit to a very recent study that showed prediction of Aβ-positivity by FDG-PET patterns (Parmera et al., 2020).

The idea of using perfusion phase Aβ-PET imaging as a surrogate of glucose metabolism is based on earlier studies comparing perfusion SPECT and FDG-PET for assessment of neuronal injury in mild cognitive impairment and AD (Schroeter et al., 2009). Although FDG-PET tends to have higher sensitivity over SPECT for detection of AD like neuronal injury at the single patient level (Bloudek et al., 2011), the patterns of alterations against controls were found to be comparable at the group level (Schroeter et al., 2009). The initial tracer uptake does not reflect Aβ burden *per se* but is rather a surrogate of cerebral blood flow (CBF), due to the high first pass extraction of ^18^F-flutemetamol and other lipophilic Aβ-PET tracers (Pike, 2009; Herholz and Ebmeier, 2011). Several studies were already able to prove this concept for different Aβ-PET ligands by showing the similarity of hypoperfusion and hypometabolism in mixed cohorts dominantly consisting of AD-spectrum patients (Meyer et al., 2011; Rostomian et al., 2011; Hsiao et al., 2012; Tiepolt et al., 2016; Daerr et al., 2017). Beyond reporting on the first CBS dataset investigated by dual-phase Aβ-PET, one novelty of our study comprises the evaluation of early-phase Aβ-PET in subcortical regions, which appeared to be important as strong involvement of the thalamus was previously reported in FDG-PET investigations on CBS (Mille et al., 2017; Pardini et al., 2019). Our results show similarly high levels of association between Aβ-PET perfusion and FDG-PET in subcortical areas when compared to cortical brain regions (Figure 2), thus revealing that early-phase Aβ-PET is able to detect commonly observed neuronal injury in subcortical areas of CBS patients. Another novelty is given by the comparison of asymmetry indices between early-phase Aβ-PET and FDG-PET. This is likewise important as CBS often presents with an asymmetric clinical phenotype and asymmetrical neuroimaging findings (Boxer et al., 2006). AI detected by FDG-PET were consistently resembled by perfusion phase Aβ-PET (Figure 3), indicating that this neuroimaging feature can be detected at a comparable accuracy for both neuronal injury assessments. Our data were robust when comparing global mean and cerebellar scaling as the commonly used approaches of relative quantification for FDG-PET and Aβ-PET perfusion imaging. However, global mean scaling indicated a superior level of correlation between FDG-PET and Aβ-PET perfusion when compared to cerebellar normalization. This finding could be related to an involvement of the cerebellum in the neurodegeneration topology of some patients with CBS or to the higher general robustness of global mean scaling. Larger cohorts will be necessary to investigate suitable and optimized pseudoreference tissues for Aβ-PET perfusion imaging in CBS.

As a limitation, we note that the optimal comparison between FDG-PET and Aβ-PET perfusion imaging would additionally comprise a normal cohort imaged with both biomarkers. Nonetheless, the unified use of FDG-PET controls also provided robust results for early-phase Aβ-PET imaging with ^18^F-florbetaben when assessed quantitatively and visually (Daerr et al., 2017). Thus, this methodology was adopted in the current investigation. Furthermore, the detection accuracy of neuronal injury patterns in the visual analysis did not suffer from the unified FDG-PET control cohort or from a potential detection gap between both modalities, since ICC revealed high levels for all intra-rater analyses. As an outlook, early-phase tau-PET imaging could also serve for detection of neuronal injury in CBS (Beyer et al., 2020), but we note that this modality needs to be evaluated in larger cohorts. Standardizations of perfusion imaging across tracers and analytic methods remain open tasks that need to be addressed by the neuroimaging communities. Currently, different recommendations of the used time-window and the used methodology (i.e., SUVr vs. R1) for different tracers hamper standardized application in clinical settings and deserve future unification (Hsiao et al., 2012; Gjedde et al., 2013; Daerr et al., 2017; Peretti et al., 2019; Ponto et al., 2019). Our data of perfusion imaging in CBS revealed a stable agreement between single 1-min frames of ^18^F-flutemetamol Aβ-PET and FDG-PET, indicating that the methodology itself is rather robust. Different spatial normalization approaches (i.e., unified versus tracer specific templates) may also have an impact on the agreement between early-phase Aβ-PET and FDG-PET, but we did not observe major differences in exploratory testing of mixed FDG/perfusion templates (data not shown). Multicenter data analyses could serve to close the gap of missing control cohorts and lacking implementation in standard software packages.

The present study demonstrates that cortical and subcortical neuronal injury can be sufficiently detected by perfusion phase Aβ-PET. The methodology is also capable to detect asymmetry of neuronal injury in CBS which provides an important neuroimaging feature in the evaluation of suspected CBS. A dual phase ^18^F-flutemetamol protocol can provide combined biomarker information on neurodegeneration and amyloid pathology, thus reducing radiation exposure and patient effort. Future studies should focus on potential temporal gaps in the detection of neuronal damage by perfusion imaging and FDG-PET.

## German Imaging Initiative for Tauopathies (GII4T)

JL, Jonathan Vöglein, Urban Fietzek, Sonja Schönecker, Georg Nübling, Catharina Prix, Kai Bötzel, Adrian Danek, Carla Palleis, Endy Weidinger, and Sabrina Katzdobler, LMU Munich, Department of Neurology.

MB, Mengmeng Song, Alexander Nitschmann, Maike Kern, Gloria Biechele, Anika Finze, Leonie Beyer, Peter Bartenstein, Stefanie Harris, Julia Schmitt, Florian Eckenweber, Simon Lindner, Franz-Joseph Gildehaus, Emanuel Joseph, Maximilian Scheifele, and Christian Zach, LMU Munich, Department of Nuclear Medicine

RP and Jan Häckert, LMU Munich, Department of Psychiatry and Psychotherapy

B-SR and Sophia Stöcklein, LMU Munich, Department of Radiology

Günter Höglinger and Gesine Respondek, Hannover Medical School, Department of Neurology

Henryk Barthel, Marianne Patt, Andreas Schildan, Osama Sabri, and Michael Rullmann, University of Leipzig, Department Nuclear Medicine

Joseph Classen, Dorothee Saur, and Jost-Julian Rumpf, University of Leipzig, Department of Neurology

Matthias L. Schroeter, Max-Plank-Institute Leipzig

Matthias Höllerhage, Technical University of Munich, Department Neurology

Alexander Drzezga, Thilo van Eimeren, Jochen Hammes, and Bernd Neumaier, University of Cologne, Department of Nuclear Medicine and Forschungszentrum Jülich

Michael T. Barbe and Oezguer Onur, University of Cologne, Department of Neurology

Estrella Morenas-Rodriguez, Jochen Herms, Sigrun Roeber, Thomas Arzberger, CH, and Frank Jessen, DZNE Munich/Bonn

Andrew Stephens, Norman Koglin, and Andre Mueller, Life Molecular Imaging

## Data Availability Statement

The raw data supporting the conclusions of this article will be made available by the authors, without undue reservation.

## Ethics Statement

The studies involving human participants were reviewed and approved by Ethics committee of the Ludwig-Maximilians-University Munich (project numbers 17–755 and 17–569). The patients/participants provided their written informed consent to participate in this study. Written informed consent was obtained from the individual(s) for the publication of any potentially identifiable images or data included in this article.

## Author Contributions

JSc writing, data analyses, and statistical analyses. CPa, CPr, EW, SK, OW, AD, and BR patient recruitment, patient evaluation, and data analyses. JSa, MU, SH, and LB PET scans and PET data analyses. AR, MS, PB, RP, CH, JL, and GH study design and conception. MB writing, drafting, and study conception. All authors added significant scientific input and intellectual content to the manuscript.

## Conflict of Interest

MB received speaker honoraria from GE healthcare and LMI and is an advisor of LMI. GH has ongoing research collaborations with Prothena; serves as a consultant for AbbVie, AlzProtect, Asceneuron, Biogen, Biohaven, Lundbeck, Novartis, Roche, Sanofi, UCB; received honoraria for scientific presentations from AbbVie, Bial, Biogen, Bristol Myers Squibb, Roche, Teva, UCB, and Zambon; and holds a patent on PERK Activation for the Treatment of Neurodegenerative Diseases (PCT/EP2015/068734). CH is chief scientific advisor of ISAR biosciences and collaborates with DENALI therapeutics. RP is on the advisory board for Biogen, has consulted for Eli Lilly and Roche, is a grant recipient from Janssen Pharmaceutica and Boehringer Ingelheim, and has received speaker honoraria from Janssen-Cilag, Pfizer and Biogen. JL reports speaker fees from Bayer Vital, consulting fees from Axon Neuroscience, author fees from Thieme medical publishers and W. Kohlhammer GmbH medical publishers, non-financial support from Abbvie and compensation for duty as part-time CMO from MODAG GmbH, all outside the submitted work. The remaining authors declare that the research was conducted in the absence of any commercial or financial relationships that could be construed as a potential conflict of interest.
