# Supplementary material for: Dual-Phase β-Amyloid PET Captures Neuronal Injury and Amyloidosis in Corticobasal Syndrome
Source: Front Aging Neurosci. 2021 May 13;13:661284. doi: 10.3389/fnagi.2021.661284 (PMC8155727; doi:10.3389/fnagi.2021.661284)
Supplement: Supplementary file 1 [file Data_Sheet_1.PDF]

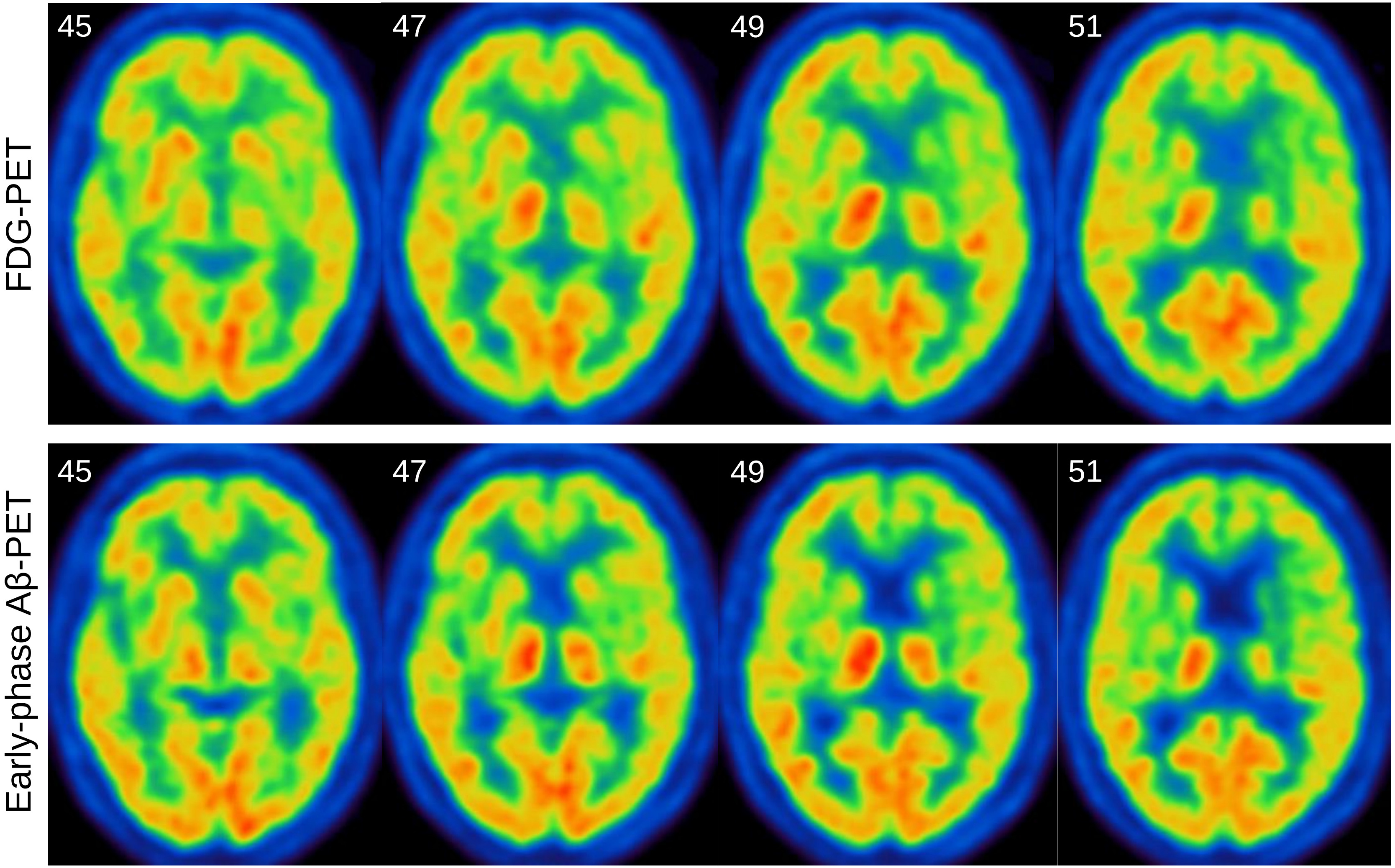

Supplemental Figure 1: Examples of the axial slices provided for the visual read. Locations of the single images represent the number of the axial slice (from a total of 109 axial slices) in the Hermes software package (FDG-PET viewer, V4.17, HERMES medical solutions AD, Stockholm, Sweden). Axial slices were selected to represent the striatum (45&47) and the thalamus (49&51).
