# Supplementary material for: Dual-Phase β-Amyloid PET Captures Neuronal Injury and Amyloidosis in Corticobasal Syndrome
Source: Front Aging Neurosci. 2021 May 13;13:661284. doi: 10.3389/fnagi.2021.661284 (PMC8155727; doi:10.3389/fnagi.2021.661284)
Supplement: Supplementary file 2 [file Data_Sheet_2.PDF]

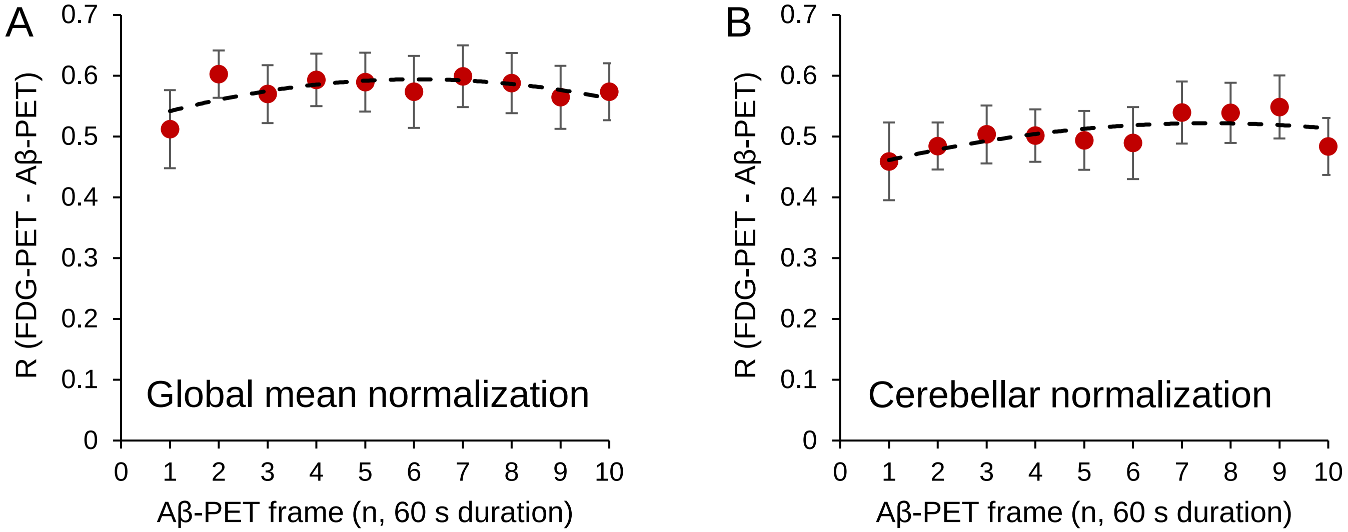

Supplemental Figure 1: In a subsample of 12 cases with one-minute frame reconstruction of early-phase Aβ-PET, SUVr values of each frame were correlated with SUVr values of FDG-PET in order to test the agreement between both modalities as a function of the early-phase Aβ-PET acquisition time. The analysis was performed for global mean normalization (A) and cerebellar normalization (B).
